# Using human pluripotent stem cell models to study autism in the era of big data

**DOI:** 10.1186/s13229-020-00322-9

**Published:** 2020-03-23

**Authors:** Ralda Nehme, Lindy E. Barrett

**Affiliations:** 1 Stanley Center for Psychiatric Research, Broad Institute of MIT and Harvard, Cambridge, MA 02142 USA; 2 Department of Stem Cell and Regenerative Biology, Harvard University, Cambridge, MA 02138 USA

**Keywords:** hPSC, ESC, iPSC, Variance, Differentiation, Sample size, Human brain, In vivo, Regulatory policy

## Abstract

Advances in human pluripotent stem cell (hPSC) biology coupled with protocols to generate diverse brain cell types in vitro have provided neuroscientists with opportunities to dissect basic and disease mechanisms in increasingly relevant cellular substrates. At the same time, large data collections and analyses have facilitated unprecedented insights into autism genetics, normal human genetic variation, and the molecular landscape of the developing human brain. While such insights have enabled the investigation of key mechanistic questions in autism, they also highlight important limitations associated with the use of existing hPSC models. In this review, we discuss four such issues which influence the efficacy of hPSC models for studying autism, including (i) sources of variance, (ii) scale and format of study design, (iii) divergence from the human brain in vivo, and (iv) regulatory policies and compliance governing the use of hPSCs. Moreover, we advocate for a set of immediate and long-term priorities to address these issues and to accelerate the generation and reproducibility of data in order to facilitate future fundamental as well as therapeutic discoveries.

## Background

“*The name ‘stem cell’ seems to me the most simple and appropriate one, because all other cells stem from it and because it is in the most literal sense the stem father as well as the stem mother of all the countless generations of cells of which the multicellular organism is later composed*” [1]

**Fig. 1:**
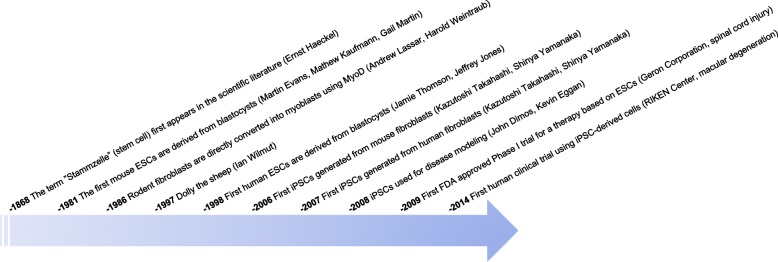
Timeline of seminal discoveries that have contributed to the current landscape of stem cell research

Since the first derivation of human embryonic stem cells (ESCs) by Jamie Thomson and Jeffrey Jones in 1998 [2] and the generation of human induced pluripotent stem cells (iPSCs) by Kazutoshi Takahashi and Shinya Yamanka in 2007 [3] (Fig. 1), combined with protocols to generate differentiated cell types in vitro, our ability to dissect basic biological mechanisms with relevance to human disease has fundamentally changed. With limited access to human tissue, neuroscientists were among the earliest adaptors of iPSC technology [4, 5] and indeed this has rapidly altered the landscape of neurodevelopmental and neurodegenerative disease modeling. While non-human model systems will continue to provide essential biological insights, there are aspects of human biology that are most accurately captured using human material. The importance of human ESCs and iPSCs, collectively called human pluripotent stem cells (hPSCs), for the study of human biology is well appreciated and the subject of numerous comprehensive reviews [6–8]. Importantly, hPSCs can be propagated indefinitely in vitro, maintaining the potential to differentiate into any type of somatic cell. Thus, hPSCs hold significant potential for understanding principles of normal human development, for the study of diverse disease mechanisms as well as for drug screening and in some cases, transplant medicine. While hPSCs by definition harbor human genotypes, these in vitro systems currently fail to capture complex gene-environment interactions that may impact development or disease in vivo. Moreover, given the protracted nature of human development, most in vitro-derived cell types remain fetal in nature which may limit their applications. As discussed in detail below, hPSC models also require rigorous attention to experimental design and understanding of their limitations before extrapolation to human in vivo biology. Compelling arguments for the use of hPSC models to study autism spectrum disorder (ASD) include limited access to tissue from the developing human brain, the prevalence of disease associated variants in non-coding regulatory regions less well conserved across species, and the lack of (or evolutionary differences between) brain structures such as the prefrontal cortex in animals that are associated with higher order cognition and function in humans and implicated in ASD pathophysiology [6, 9–11]. Moreover, recent human genetic studies combined with RNA-sequencing analyses from the human brain report that a majority of genes mutated in ASD are expressed in the excitatory and inhibitory neuronal lineages of the cortex during prenatal development [12]. Furthermore, single-nucleus RNA-sequencing studies from ASD patient cortical tissues nominated upper-layer excitatory cortical neurons along with microglia as cell types preferentially affected in ASD [13]. These findings make in vitro differentiated brain cell types including excitatory neurons, inhibitory neurons, and microglia, which more closely correlate with prenatal rather than postnatal cell states at molecular and functional levels [14–16], particularly useful for studies of ASD. Such cell types can be generated from hPSCs by either organoid (three dimensional) or monolayer approaches. Organoid approaches generate different cell types [14, 17], and thus facilitate studies of the interaction of multiple cell types, and consequently, cell circuitry level mechanisms but a limited number of studies have utilized organoids for the study of ASD [18, 19]. Conversely, many monolayer approaches have been optimized to give rise to more reproducible, homogenous populations of cells (such as excitatory neurons [16, 20]) across individuals and are thus well suited for the investigation ASD-related mechanisms in individual cell types. Thus, by acquiring or inducing ASD-relevant genetic perturbations in hPSCs and generating brain cell types in vitro, investigators can now address mechanistic questions in a tractable system with relevance to the developing human brain.

ASD is a neurodevelopmental disease characterized at its core by impaired social/communication skills and restrictive/repetitive behavior, with high heritability and a prevalence of 1–1.5% [21, 22]. However, as the term “spectrum” indicates, patients exhibit a wide range of behavioral phenotypes and may present with other neurological or non-neurological comorbidities (e.g., epilepsy, intellectual disability, gastrointestinal symptoms). This phenotypic complexity is paralleled by complexity in the underlying genetic architecture, with contributions from rare structural variants of large effect (e.g., 16p11.2 or 22q11.2 duplication or deletion), rare coding variants of large effect (e.g., *CHD8*, *SCN2A*), and common variants associated with small effect sizes [21, 23]. Importantly, large-scale exome sequencing studies of ASD have now provided investigators with over 100 high-confidence autism susceptibility genes to interrogate [12, 24, 25] and genome-wide association studies have identified several significant loci [22]. However, it is estimated that common variants, a majority of which remain unknown, account for up to half of overall ASD risk, with 5–30% of risk coming from rare variants and the remaining risk not yet defined [26–28]. This means that only a minority of ASD patients (~ 25%) are found to carry known or candidate sequence or structural variants, and a majority (~ 75%) are classified as nonsyndromic idiopathic [21]. Indeed, individual ASD risk genes have not been reported in more than 2% of patients [26], and even in patients harboring a rare, large effect de novo variant, common variation is thought to contribute additively to risk [28], making common variation and its influence on phenotypic presentation an important consideration in all forms of the disease. For hPSC models of autism, this means that a substantial amount of genetic risk remains undefined in donor samples. In addition to identifying all genetic drivers of ASD, how each variant interacts with a patient’s unique genetic landscape and ultimately with environmental factors to contribute to disease presentation remain key unanswered questions in the field. Thus, while big data has yielded groundbreaking insights into ASD, it has also illuminated the substantial complexities involved in optimally employing hPSC models to study disease mechanisms. Specifically, hPSC models (i) are highly sensitive to genetic and experimental variance which can complicate detection and reproducibility of phenotypes in vitro, (ii) require new frameworks for study design based on the underlying genetic complexity, (iii) have both known and unknown divergence from the human brain in vivo, and (iv) are subject to a complex regulatory landscape which governs access to both materials and datasets. Based on current hPSC tools, coupled with emerging data in autism genetics and the human brain, we advocate for further investment in several critical areas to improve the detection, reproducibility, and applicability of relevant phenotypes in hPSC models of ASD and to accelerate future discovery.

## Common sources of variance

“*The whole idea of a stereotype is to simplify. Instead of going through the problem of all this great diversity – that it’s this or maybe that – you have just one large statement; it is this*” [29]

In order to facilitate cross-study comparisons in ASD, it is essential not only that investigators understand the many sources of variance and how they may impact biological conclusions, but that publications clearly define and report the specific conditions utilized in hPSC studies that may contribute to variance. Below, we discuss two broad categories of variance. We begin with a brief discussion of common *unwanted* sources of variance such as culture induced genetic or epigenetic changes. We then expand on two *wanted* sources of variance including sex and ancestry diversity, where efforts to minimize variance have contributed to an overrepresentation of XY lines of European ancestry in hPSC research.

### Unwanted variance

The genetic basis of ASD has obvious implications for disease modeling. While rare variants of large effect are more likely to yield penetrant in vitro phenotypes, common variants of small effect are likely to translate into more subtle phenotypes in vitro*.* As such, the former are expected to be more robust against the many sources of variance that impact hPSC models of ASD compared with the latter. It is important to note however, that even when studying syndromic ASD with hPSC models, variable penetrance, pleiotropy, and phenotypic heterogeneity mean that the same variant may still lead to the expression of different phenotypes in vitro depending on a host of technical or biological factors. For example, Fragile X syndrome (FXS) is a leading monogenic cause of ASD, driven by loss of the *Fragile X Mental Retardation 1* (*FMR1*) gene. Despite being driven by loss of a single, well-characterized gene, different studies using hPSC models have drawn opposing conclusions with regard to the impact of *FMR1* loss on cellular function, likely due in part to key differences in the experimental paradigms across studies. Sheridan et al. [30], Doers et al. [31], Boland et al. [32], and Zhang et al. [33], each utilized FXS patient and control iPSC lines to generate neurons in vitro. While Sheridan et al. and Doers et al. detected decreased neurite outgrowth in patient cell lines compared to controls [30, 31], Boland et al. detected increased neurite outgrowth [32] and Zhang et al. reported typical neuronal morphology in cells from both groups [33]. These study designs differed in the specific neuronal cell types generated in vitro (i.e., driven by developmental patterning versus transcription factor overexpression), the culture conditions under which the neurons were maintained (i.e., human neurons cultured alone or mixed with murine brain cell types) and the time-points of analyses (i.e., during differentiation or in post-mitotic neurons). Along similar lines, Mariani et al. and Marchetto et al. both utilized iPSC models of nonsyndromic idiopathic ASD and while Mariani et al. reported increased synaptogenesis in patient cell lines compared to controls, Marchetto et al. reported decreased synaptogenesis in patient cell lines compared to controls [18, 34]. Indeed, there is little agreement in the literature on ASD-relevant cellular phenotypes in vitro, and whether discrepancies are due to true biological variation in ASD or to technical variation. Importantly, there are many factors that have the potential to profoundly impact the presentation of ASD phenotypes in vitro regardless of the genetic basis of the disease, although the landscape becomes increasingly complex when considering idiopathic compared to syndromic disease. These include differences in donor cell type, age and method of hPSC derivation, culture-induced genetic or epigenetic variation, human genetic variation, method and timing of gene manipulation, cell type(s) and cell ratios generated by distinct in vitro differentiation paradigms, the genes or chemicals used to drive in vitro differentiation, culture conditions, and time-point(s) of analysis (Table 1).

**Table 1:** Examples of common sources of variance in hPSC models

Common source of variance	Examples/considerations
hPSC derivation	ESC versus iPSC; iPSC reprogramming methodology, donor cell type, donor age
Culture induced genetic or epigenetic changes	Trisomies, CNVs, and SNVs acquired during reprogramming or with continued passage; X chromosome status in XX cell lines
Human genetic variation	Influence of genetic background on expression of phenotypes; selection of related or unrelated controls, sex
Method and timing of gene manipulation	Constitutive versus inducible; transient versus stable
Cell types/ratios	Monolayer versus organoid; pure versus mixed brain cell types; different subtypes of excitatory neurons, inhibitory neurons, and glia
Differentiation paradigm	Developmental patterning versus transcription factor overexpression; batch effects across differentiations
Culture conditions	Naïve versus primed pluripotent conditions; coculture with cells from other species, with or without genetic manipulation; substrate, density, and media composition
Time-point of analysis	Pluripotent, progenitor, or post-mitotic cell stages

Expanding on one example, culture induced genetic changes are unwanted sources of variance that frequently occur in vitro. The events that take place between fertilization and implantation during normal embryonic development in vivo are exquisitely timed and executed, with only transient passage through each cellular state. However, ESCs isolated from the inner cell mass of pre-implantation blastocysts and iPSCs reprogrammed from somatic cell types are both maintained in a proliferative state in vitro that lasts indefinitely. Thus, both cell types have the opportunity to undergo significant genetic and epigenetic drift or divergence over time. While different in vitro environmental conditions such as the use of naïve versus primed culture medias or single-cell isolation can influence the propensity of cells to undergo genetic or epigenetic changes, all hPSC lines undergo continual cell division and thus may acquire alterations that influence their behavior. Specifically with regard to culture conditions, previous studies have shown that naïve culture conditions can contribute to chromosomal abnormalities in hPSCs [35] and thus require particularly close attention when being considered for disease modeling. Overall, genetic changes may come in the form of trisomies, copy number variations (CNVs), or single nucleotide variations (SNVs), with those conferring a selective growth advantage able to rapidly permeate through culture dishes. While trisomies are readily detectable, the gold-standard G-band karyotyping analysis for assessing cell line integrity has a lower limit of detection of around 5 Mb and high density SNP genotyping arrays typically do not capture CNVs smaller than 0.5 Mb. This means that small CNVs and SNVs may be missed with the most commonly employed technologies for analyzing cell line integrity. Importantly, mosaic mutations, including deletions and duplications, may arise in a small subset of cells that either confer a selective growth advantage and take over the culture, or remain at a stable cell fraction. Depending on the extent of mosaicism, these mutations may sit below the level of detection in a majority of assays. One study employing whole exome sequencing (WES) of 140 hESC lines revealed that 5 lines carried mutations in *TP53*, which conferred a selective growth advantage upon continued passage, and similar results were obtained through analysis of published RNA-seq datasets using other hPSC collections [36]. In some cases, the same parental cell line used in different studies from different laboratories showed variability in the presence of *TP53* mutations [36]. While 5/140 lines (3.57%) is a small fraction of cell lines with *TP53* mutations, other gene mutations could similarly confer a selective growth advantage and gene-level analyses are not standardly employed in hPSC studies. This result has implications not only for the clinical utility of hPSCs, but also for studies of basic disease mechanisms, where the presence of single-gene mutation could significantly impact hPSC phenotypes. hPSCs assumed to be isogenic are thus unlikely to remain truly isogenic over time and the same parental cell line utilized by two different laboratories may have relevant genetic differences with the potential to influence molecular and cellular phenotypes that go undetected due to their size or percent mosaicism. It is therefore critical to improve and standardize methods to assess genomic integrity of hPSC lines in order to ensure that any reported phenotypes are not caused by underlying genotypic abnormalities. Importantly, additional studies are needed to assess the influence of specific culture conditions on the propensity of hPSC lines to acquire abnormalities.

While we would generally argue for minimization of variance in hPSC models of ASD such as the example given above, we have selected two sources of variance, ancestry and sex, which have profoundly shaped the landscape of hPSC lines selected for study of ASD and inadvertently biased our biological insights toward a minority of ASD patients.

### Wanted variance: human genetic diversity

As the use of genome-wide association studies (GWAS), whole genome sequencing (WGS), and WES rapidly expand across different populations, we have become increasingly aware of the profound degree of human genetic variation underlying population diversity. For example, analyses of data from 2504 human genomes estimate around 18.4 Mb of structural variants per individual [37]. Analyses of WES data from 60,706 individuals estimate one variant for every eight bases of the exome [38]. Further, analyses of WGS data from 816 parent-child trios suggest that each individual carries an average of 45 de novo mutations [39]. Thus, the question of what makes an individual an appropriate control becomes more complex, and genetic differences unrelated to ASD have the potential to influence cellular phenotypes in vitro. Similarly, variants have not been systematically studied across different individuals to understand how genetic background may influence expression of disease phenotypes. The concept that normal human genetic variation (e.g., not associated with a specific disease diagnosis) across individuals contributes to variation in cellular models is borne out by large-scale phenotyping studies of iPSCs. Indeed, the human induced pluripotent stem cells initiative generated, genotyped, and phenotyped 711 iPSC lines from 301 health individuals and found that anywhere from 5–46% of variance in genome-wide assays, protein immunostaining assays, and cellular morphology assays was due to differences across individual donors [40]. Additional sources of variance included biological or experimental factors such as assay batch effects, cell line passage number, and karyotype abnormalities [40], as discussed above. Thus, the authors conclude that genetic variation from healthy individuals impacts molecular and cellular phenotypes in iPSCs [40]. Similarly, in a study of 317 iPSC lines from 101 individuals, roughly 50% of the variability in gene expression was reportedly due to donor specific differences [41], reinforcing the notion that normal genetic variation can have a sizeable impact on hPSC phenotypes.

**Fig. 2:**
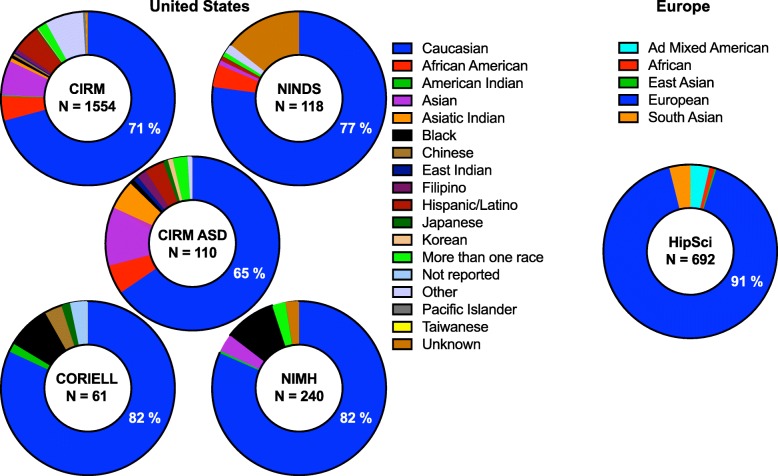
Examples of large iPSC collections in the USA (left) and Europe (right) analyzed by race. Percentage of samples from a collection categorized as Caucasian or European indicated in blue. Total number of iPSC lines in a collection shown in center of each collection

Adding to the complexity, it has become increasingly clear that human genetic studies have a diversity problem, specifically with regard to the overrepresentation of European ancestries [42, 43]. Analyses by Martin et al. [43] highlight the fact that while individuals of European descent make up 16% of the population worldwide, they account for around 79% of participants in GWAS [43]. This disparity translates into genetic prediction accuracy 4.5-fold lower in African individuals and 2.0-fold lower in East Asian compared to European individuals with these datasets [43]. Thus, our genetic insights into many diseases including ASD are weighted toward a minority of the global population. Not unexpectedly, this bias toward European ancestries is also paralleled in the existing iPSC collections in the USA and elsewhere, including current ASD collections. Analyses of iPSC lines available through the California Institute of Regenerative Medicine (CIRM) iPSC Repository at FujiFilm Cellular Dynamics as of September 2019 reveals that 70.85% (1101/1554) of the collection is Caucasian and similarly, 65.45% of their ASD collection is Caucasian (72/110; Fig. 2). This bias is also observed with iPSC lines available through other collections in the USA (i.e., the NIGMS and NIA collections at Coriell, the NINDS collection and the NIMH collection) as well as in Europe (i.e., HipSci), all of which include a variety of normal and disease lines (Fig. 2).

Thus, individual investigators utilizing hPSC models of ASD and other diseases may find themselves in a difficult position, where ancestry, sex, and age matched case-control cohorts are the current best practice to minimize assay variance. However, there is also urgent need to define which differences in genetic background meaningfully contribute to expression of phenotypes, and to utilize increasingly diverse collections of cellular resources as global genetic datasets expand. Indeed, while iPSC collections currently roughly mirror the ancestries represented in human genetic datasets, these datasets are now expanding to achieve greater diversity through efforts such as the Stanley Global Neuropsychiatric Genetics Initiative (Broad Institute) and the All of Us Research Program (NIH). Parallel diversification in iPSC collections would allow the field to capitalize on functional assessment of variants identified in non-European ancestries that may provide key mechanistic insight, to leverage genetic diversity toward assessment of pathological versus non-pathological variants and to minimize risk that future discoveries exclude a majority of the global population. The balance struck between maintaining homogeneity in ancestry to minimize variance and increasing genetic diversity to better represent the global population has significant scientific as well as societal and ethical implications. The bottom line is that if investigators do not actively generate and utilize increasing numbers of iPSC lines from diverse genetic backgrounds, the default setting will continue to be hPSC models of ASD and other diseases from almost exclusively European ancestries.

### Wanted variance: XX hPSC lines

**Fig. 3:**
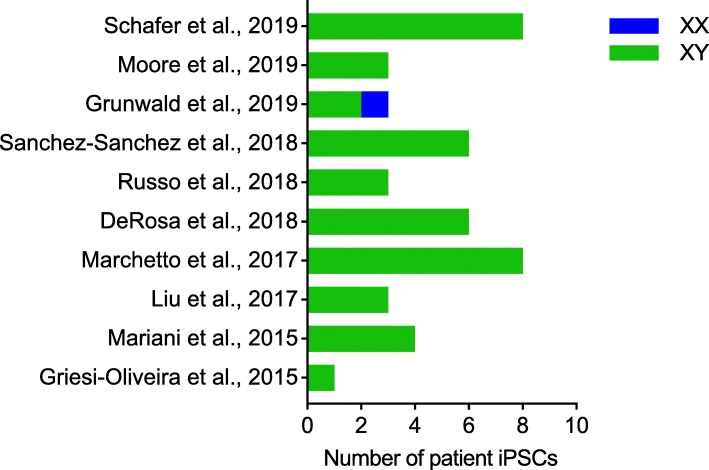
Examples of studies utilizing iPSC models to study nonsyndromic idiopathic ASD [18, 34, 49–56]. Studies are ordered by year with the most recent at the top and include the total number of XX and XY patient iPSCs used in the bar chart

Aberrant X chromosome inactivation (XCI) mediated by the long non-coding RNA *XIST* is an important example of a culture-induced epigenetic change in hPSC lines in vitro with relevance to ASD research. While early XX embryonic cells in vivo exist in a pre-XCI state, somatic XX cells in vivo normally have one active X chromosome and one inactive X chromosome [44]. By contrast, XX hPSC lines can show erosion of XCI in vitro through loss of *XIST*, leading to complex scenarios including complete reactivation (two active X chromosomes), heterogeneous populations of inactivation and reactivation and partially eroded intermediates [45], and this can be influenced by the use of naïve versus primed culture conditions [46]. The sex of hPSC lines is obviously a critical consideration for studies of X-linked neurodevelopmental diseases and indeed, Mekhoubad et al. showed decreased *XIST* expression in XX lines over extended passage in vitro and parallel loss of cellular phenotypes in Lesch-Nyhan syndrome patient iPSCs [47]. While a majority of known ASD risk genes do not reside on sex chromosomes, there is clear sex-specific modulation of penetrance [45] and importantly, the lack of ability to readily monitor or manipulate XCI in vitro may contribute to the preferential use of XY cell lines over XX cell lines in ASD. Indeed, out of ten recent papers we identified utilizing iPSC models of nonsyndromic idiopathic ASD, we were surprised to find that 97.8% (44/45) of ASD patient iPSCs collectively utilized in these studies were XY, with only 2.2% (1/45) XX ASD patient iPSC employed (Fig. 3). One could argue this is due to males being more frequently affected by ASD than females, but females are still diagnosed with ASD at a rate of approximately 1/189 (compared with approximately 1/42 males) [48]. It is important to note that a majority of large iPSC collections discussed in Fig. 2 are roughly sex balanced, and 31% of CIRM’s ASD iPSC collection is XX (34/110), which means that a lack of available XX iPSC lines is not driving the disparity in their utilization. As discussed above with regard to European ancestries, if investigators do not recognize and address sex bias in hPSC models of ASD, it is likely that XY cohorts will continue to dominate studies, and a failure to study XX lines may also limit our ability to understand how sex differences intersect with ASD liability.

We elected to focus on human genetic diversity and sex balance above, specifically because efforts to minimize these sources of variance have driven a disparity in research involving hPSC models of ASD. Looking forward, we advocate for a more conscious approach to ancestry and sex, which will likely require the development of methods to monitor and/or manipulate X chromosome status in vitro and investment in global collection efforts to increase the numbers of iPSC lines generated from diverse genetic backgrounds. While we recognize the contradiction in arguing to increase variance in hPSC models given the complexities shown in Table 1, we view this as both an ethical obligation as well as a strategy that can be leveraged in the future to gain insight into the contribution of factors that may diverge across different populations affected by ASD. Moreover, minimizing *unwanted* sources of variance is key to facilitating the introduction of biologically meaningful *wanted* sources of variance like ancestry and sex into hPSC models of ASD.

## Design of appropriately powered studies

“*In theory, there is no difference between theory and practice, while in practice there is*” [57]

Sound statistical power calculations have fueled the success of large-scale genetic analyses [28, 58, 59]. However, experimental biological studies by and large lack statistically informed standards of how many cases and controls are sufficient in order to draw conclusions given different underlying genetic architectures. While tens of thousands of samples are required to achieve sufficient statistical power in genetic studies of ASD [60], it is currently unclear how many samples are needed for different iPSC-based studies. Due to the nature of biological experimentation, the ideal number of samples will vary depending on (1) the specific differentiation protocol used, and the variance/reproducibility across biological replicates for that particular protocol; (2) the end-point assay (for example, RNA-sequencing versus proteomics versus electrophysiological readout), each with different degrees of technical variance that can change depending upon experimental design; and (3) the nature of the ASD variants being investigated (for example, syndromic versus non-syndromic) and relatedness of the cohorts, as further discussed below. While most investigators agree that study designs used for inbred mouse strains are not equally appropriate for drawing conclusions using hPSC models, cost largely prohibits the scale that power calculations may predict are necessary to identify small effect sizes among unstratified cohorts. Moreover, a majority of the available patient iPSC lines for studying idiopathic ASD are not connected to genotyping or sequencing data, nor are they connected to patient phenotyping data, making it difficult to rationally select or group cell lines for study. Thus, studies may be restricted to the detection of very large effect sizes and highly sensitive to technical or experimental variation, leading to challenges in data consistency and reproducibility across laboratories. Below, we discuss the ideal scenario for study design, followed by practical strategies to decrease sample size in recognition that the ideal scenario is largely cost prohibitive for a majority of laboratories and institutions.

### An ideal scenario

An ideal practice for study design is to first run power calculations based on pilot experiments, and compute the variance for each specific experimental system. The needed sample size can then be estimated based on the amplitude of the desired effect. The larger the variance in the system, the bigger the needed sample size. Greater variance is typically associated with idiopathic versus genetically stratified cohorts, thus, in general, one would expect a larger sample size to be necessary for these studies [61]. For example, large, penetrant CNVs such as the 22q11.2 deletion often affect the expression of many genes within the CNV boundary [62]. Deletion of one copy of these genes should result in a 1.5 to 2-fold reduction in gene expression. If differential gene expression analyses between cases and controls fails to detect a reduction in the expression of these genes that passes a significance threshold (typically, false discovery rate (FDR) < 5%), then the study is unlikely to be sufficiently powered to detect any changes of that size. If, on the other hand, it does pass significance, the study is not only powered to detect changes in these genes, but it is also sufficiently powered to detect changes in other genes that are of a similar effect size, or larger. Notably, a study of sensory neurons generated from 100 unique, healthy iPSC donors calculated a between-sample variance of 0.37 (as determined by the coefficient of variation (CV)), compared with 0.23 in postmortem dorsal root ganglion tissue, leading the authors to conclude that gene expression is more variable in in vitro cultures compared with the relevant primary tissue [63]. They further determined that 24.7% of variance was explained by the batch of the neuronal differentiation, while 23.3% of the variance was explained by the donor iPSC line of origin, supporting the notion that differentiation conditions as well as genetic variation are significant contributors to assay variance. The authors predicted that sample sizes of 20–80 iPSC lines from unrelated individuals would be required to study variants with 1.5- to 2- fold allelic fold changes, while hundreds of lines would be necessary to discover quantitative trait loci (QTLs) with more modest effects [63]. However, nearly all published iPSC studies of idiopathic nonsyndromic ASD have utilized sample sizes < 10, with roughly half of the studies utilizing sample sizes ≤3 (Fig. 3), reflecting the practical realities associated with scaled experiments of this nature.

### Strategies to reduce sample size

While new technologies and innovations may drive down the per sample cost of experiments using hPSC models in the future, there are several strategies that can be used at present to decrease sample size. First, studies using related controls such as trio or quad designs that minimize genetic variation between ASD probands and controls may require smaller sample numbers compared with studies using unrelated controls in order to detect significant signal above background. Indeed, the selection of control individuals is equally as important as the selection of ASD patients in study design, and can influence the degree to which phenotypes are detected (or not detected) in a given assay [61]. As discussed below, this will require greater access to family-based ASD hPSC collections which are already widely utilized in genetic studies, to achieve statistical power with a more tenable number of samples. For example, of 1554 iPSC cell lines currently in the CIRM collection discussed in Fig. 2, 45 are derived from unaffected family members, suggesting such samples do exist for some diseases albeit in very small numbers at present. Second, increasing the number of experimental and biological replicates typically augments the statistical power, with better outcomes expected from using a larger number of donors whenever possible, over independent clones from the same donor [64]. Third, the inclusion of isogenic engineered cohorts to validate findings from patient-based cohorts can further demonstrate causal relationships between individual genetic variants and phenotypic outcomes [64]. Fourth, when utilizing small cohorts or those with incompletely defined genetic underpinnings, reporting results for each individual donor as opposed to averaging across diverse samples may identify trends that vary in magnitude or directionality for specific subsets of ASD patients but are nonetheless relevant. Indeed, the application of standard statistical thresholds such as FDR < 5% may not be appropriate for underpowered, heterogeneous cohorts, which may instead benefit from more “within patient” analyses. Finally, power calculations can be leveraged not just to predict how many samples a given study should use, but also to clearly define the magnitude of effect that a study is powered to detect, and what may be missed, from a given sample size.

## Divergence from the human brain in vivo

“*Remember that all models are wrong; the practical question is how wrong do they have to be to not be useful*” [65]

As discussed above, the prenatal cortex is thought to be a critical structure for ASD pathogenesis, making in vitro differentiated brain cell types particularly well-suited for capturing relevant molecular and functional features [14–16]. However, in vitro models are known to diverge from their in vivo counterparts in several key areas and our knowledge of the developing human brain remains incomplete. While the utility of in vitro models to study in vivo development has been the subject of numerous reviews [17, 66, 67], we have selected two key points to discuss with relevance to ASD research. First, there are aspects of cell composition, cell maturation, and organization of current in vitro cultured preparations that are relevant to ASD but known to diverge from the in vivo norm and thus must to be taken into consideration when extrapolating to disease mechanisms, and/or further improved upon with new technologies. Second, molecular mechanisms underlying normal human brain development in vivo remain incompletely understood, meaning additional differences are likely to emerge from ongoing exploration of the developing human brain that must be continually incorporated into interpretations of ASD mechanisms. In both cases, the question is not only how similar are in vitro and in vivo systems, but also, to what extent and in what dimensions is sameness required.

### Known divergence: cell composition, maturation, and organization

hPSC-derived brain cell types generated by different approaches have been shown to share some of the properties of their native counterparts. Similarities include, for instance, the general neuronal fate (whether excitatory or inhibitory), as determined by the expression of a subset of molecular markers, along with predicted electrophysiological properties of neurons [14, 16, 20]. However, while organoid approaches and monolayer cultures can harbor an array of different cell types, these schemes often give rise to cell ratios that deviate from those found in the human brain [17, 67]. This is a particularly relevant consideration for the study ASD, which many hypothesize results from an excitatory/inhibitory imbalance [12]. Thus, careful attention must be paid to the influence of cell composition on non-cell-autonomous molecular and functional phenotypes. Additionally, not all cell types are readily generated in culture. Brain features such as vasculature (endothelial cells), myelination (mature oligodendrocytes), and microglia (which arise from mesoderm rather than ectoderm) are typically absent from forebrain organoids or monolayer preparations [17, 68]. These cell types may be critical mediators of the maturation of, and communication between, excitatory and inhibitory neurons more commonly implicated in ASD phenotypes. Importantly, lamination (the organization of specific cell types into layers) is not conserved in vitro [17, 66], and thus aspects of long-range connectivity may not be readily detectable with current human cellular models. Despite these limitations, ongoing innovations hold great promise for further improvement of in vitro culture systems. For instance, there are now protocols for generating relatively pure populations of excitatory [20] and inhibitory neurons [69], which can be cocultured at defined ratios. Missing cell types (such as microglia) have also been derived independently [70, 71] and added to brain cell cultures [72], and bioengineering approaches could be used to generate scaffolds that separate cells into distinct cortical layers [73]. Vascularized brain organoids have also been established by fusing neural progenitor cell spheroids with endothelial cell spheroids with the addition of mesenchymal stem cells [74]. Similarly, organoid vascularization has been achieved through ETV2 induction [75], coculture with human umbilical vein endothelial cells (HUVECs) [76] or mesodermal progenitors [77]. Moreover, examining more than one cell type and differentiation stage increases the likelihood of identifying relevant phenotypic and molecular differences in cells derived from individuals with ASD in the absence of known “cells of origin.” A good example of this comes from the Alzheimer’s disease field, where investigators have begun to probe the effects of single mutations like APOE4 using in vitro derived neurons, astrocytes, and microglia in parallel [78], in order to obtain a more comprehensive view of cell-type specific defects for a given genetic perturbation. As discussed above, upper-layer excitatory cortical neurons, inhibitory neurons, and microglia may be particularly important cell types for the study of ASD. Importantly, all of the strategies discussed above are feasible with existing technologies and can be used to address specific shortcomings with relevance to ASD.

### Unknown divergence: in vivo fidelity

Recent large-scale studies have generated critical insight into transcriptional and epigenetic landscapes of the developing human prefrontal cortex through efforts like the BrainSpan Consortia and PsychENCODE [79–81]. This has begun to allow investigators to benchmark the identity and maturity of in vitro derived cell types against current knowledge of the developing human brain and provide a more realistic understanding of which native cell types of the derived cells correlate most strongly with, and at which developmental stage. However, the sheer complexity of brain regions composed of billions of cells orchestrating higher order cognition means critical knowledge gaps remain. For example, based on current knowledge, can we definitively say what molecular or functional features are required to call a cell an inhibitory neuron, excitatory neuron, or astrocyte in vitro and in vivo? How many sub-classifications of each major brain cell type exist and what are their key molecular and functional distinctions? Given the protracted course of normal human brain development in vivo, do we have the resolution to assess which features may be lost or aberrantly expressed by the accelerated and limited nature of in vitro development? Moreover, to what extent does an experimental system actually need to match the human in vivo setting in order to facilitate important biological insight? With these questions outstanding, studies of ASD variants using distinct differentiation protocols to generate “excitatory neurons” may actually be examining different subtypes of cells or developmental stages and reach different conclusions with regard to molecular or cellular phenotypes, complicating cross-study comparisons if investigators do not explicitly frame their conclusions in the context of their experimental system. Resolving some of these questions will require the acquisition of more complete datasets throughout human brain development that expand upon the existing transcriptional and epigenetic profiles and further incorporate proteomic, morphological, and functional metrics, alongside thoughtful discussion of what insights can be garnered from model systems. While post-mortem studies may be useful for later-onset neuropsychiatric diseases like schizophrenia, fetal tissue is a critical component of ASD research given the timing of disease pathogenesis. However, on June 5, 2019, the federal government halted human fetal tissue research by NIH employed scientists, and the ability of investigators at academic institutions to use NIH funding for human fetal tissue research remains uncertain with the addition of new review processes.

Collectively, it is essential to advocate for continued investment in both human fetal tissue research as well as additional or improved methodologies for generating and studying differentiated brain cell types with relevance to ASD in order to fully capitalize on emerging human genetic data and translate that into biological and clinical insight. However, investigators must also bear in mind that in vitro model systems will always fall short of the human brain in vivo, and continually consider the degree and manner in which it is acceptable or unacceptable for a system to fall short for studies of ASD.

## Regulatory considerations for hPSC collections

“*The large print giveth and the small print taketh away*” [82]

While tremendous progress has been made in the development of approaches to generate increasingly accurate human cellular models, the regulatory framework necessary to support this growth has lagged behind and is only now beginning to catch up. A majority of the early hPSC generation efforts were taken on by individual laboratories and these collections often have restrictions in the consent forms limiting their use and whether the resulting cell lines and datasets can be shared or deposited in the public domain. This issue applies not only to hPSC models of ASD, but to many diverse hPSC resources. While regulatory considerations receive little attention from the scientific community, they can have a profound impact on scientific progress. In order to maximize these valuable resources for ASD, the design of future collection efforts requires input from donors and their families, physicians, administrators, geneticists, and investigators.

As investigators increasingly turn to large, multi-center and multi-national collaborations in order to meet statistical power requirements and cohort diversity ideals as discussed above, they remain bound by consent forms and regulatory and compliance frameworks that can limit the ability to share data and cell lines to ensure that research using the samples and/or data is consistent with donor consent. Indeed, investigators must meet requirements from funding agencies, governmental agencies, and publishers, as well as operate within compliance and regulatory frameworks set by individual institutions based on consent forms, material transfer agreements (MTAs), collaboration agreements, and Institutional Review Boards (IRBs). The review process that balances data-sharing ideals and the protection of human subjects for projects involving hPSCs includes coordination between these groups, and regulations may vary across organizations and institutions. For example, studies funded by the National Institutes of Health (NIH) in the USA are subject to the genomic data sharing (GDS) policy, requiring data deposition in repositories such as dbGAP (database of Genotypes and Phenotypes), while cell line collections originating in European countries are now subject to the GDPR (General Data Protection Regulation), designed to protect the data and privacy of European citizens, and can limit data deposition to the EGA (European Genome-phenome Archive) [83], complicating the ability of the field to share and publish valuable hPSC resources. Sample and data use agreements are typically required prior to cell line sharing and data distribution, and can be difficult to finalize, stalling publication, and resource dissemination. Streamlining these processes is especially important, given the need for iPSC collections from more diverse ancestries, which will likely require global collection and distribution of donor samples.

Some newer hPSC collections, mindful of the limitations and obstacles to broad use and distribution of lines derived through previous efforts, are striving to simplify consent forms and maximize the ability to disseminate data and reagents. For example, registries such as the NIH human embryonic stem cell registry (https://stemcells.nih.gov/), the European human pluripotent stem cell registry (hPSCreg; https://hpscreg.eu), and the Korean stem cell registry (http://www.nih.go.kr/) catalog available hPSC lines along with relevant biological information and metadata for each cell line [84, 85]. Available data often includes donor information (e.g., age, sex, disease state), cell line information (e.g., derivation method, culture conditions), and in some cases, basic cell line characterization (e.g., marker expression, differentiation potency), and genotype (e.g., individual mutations or genetic modifications). Notably, hPSCreg additionally documents pertinent legal information such as data about ethical standards for cell sourcing, privacy protection, and any restrictions on the use of cell lines for specific research applications [84]. Stem cell repositories and biobanks such as CIRM, Coriell, the European Bank of induced pluripotent Stem Cells (EBiSC), the Human iPSC Initiative (HipSci), WiCell Research Institute, and the NIMH Stem Cell Center at Rutgers University also aim to maintain, characterize, bank, and distribute hundreds of hPSC lines [85–87] (Table 2) with fewer restrictions. Of note, from the studies of nonsyndromic idiopathic ASD using iPSC lines discussed above in Fig. 3, only one study used iPSCs from a public repository [49] and the remaining were reprogrammed by individual laboratories, suggesting that public repositories are not yet widely utilized in published ASD studies. It is therefore important to ascertain whether this is for historic reasons (e.g., laboratories initiated studies prior to the availability of relevant iPSCs) or whether aspects of existing ASD collections do not meet study needs (e.g., laboratories reprogramed their own samples because of a need for phenotyping data not found in current repositories).

**Table 2:** Examples of large hPSC repositories around the world

Repository	Website
Coriell Institute Stem Cell Biobank	https://www.coriell.org
CIRM via Fujifilm	https://www.cirm.ca.gov via https://fujifilmcdi.com
NYSCF	https://nyscf.org
WiCell	https://www.wicell.org
NIMH Stem Cell Center at Rutgers University	https://www.rucdr.org/stem-cell
EBiSC	https://ebisc.org
HipSci	http://www.hipsci.org
ECACC European Collection of Authenticated Cell Cultures	https://www.phe-culturecollections.org.uk/collections/ecacc.aspx
CellBank Australia	http://www.cellbankaustralia.com
Riken Bioresource Center	https://cell.brc.riken.jp/en

Despite the challenges and restrictions associated with early hPSC collections, the establishment of new hPSC registries and repositories, coupled with a focus on streamlined consent forms and legal documentation for current and future sample collections should greatly facilitate the usability and shareability of hPSC lines and data in the years to come. Specifically with regard to ASD research, there are several features that may be key to include in future collections, and it is essential this conversation includes input from all stakeholders. First, incorporating re-contactability into donor consent forms in anticipation that ASD patient phenotyping may undergo further evolution and refinement in the future. Indeed, some argue that ASD should not be a single diagnosis, but instead should be further subdivided into more biologically meaningful categories [88]. Given the longevity of hPSC collections, re-contactability would allow existing cellular resources to continue to provide meaningful insights as clinical perspectives on ASD evolve, and avoid a scenario where refined diagnostics necessitate new and expensive hPSC collection efforts. Second, given limited knowledge of common variants in ASD, family-based collections such as trio or quad designs, as discussed above, is especially important to account for unknown genetic risk and minimize assay variance. Third, as with all hPSC collections, the ability to generate and share genomic data is an important component of new sample collections. Overall, regulations governing individual hPSC lines and datasets should be a critical consideration in the design of hPSC studies, as they will dictate key downstream steps such as publication and the ability of other laboratories to analyze and replicate datasets.

### Looking forward

For exceedingly practical reasons, the ASD field overall has focused on rare, highly penetrant mutations involving genes of known function, primarily utilizing XY hPSC lines of European ancestry. While this approach has provided, and will continue to provide, fundamental insight into the biology of ASD, continuing solely on this path will both fail to meet the global disease burden and fully address the complexities of the disease. Looking forward, we advocate for further investment in several key immediate and long-term priorities that require multidisciplinary involvement. First, a conscious approach to sex and ancestry in ASD studies using hPSCs, including improved methods to monitor and manipulate X chromosome status in vitro and increasing numbers of iPSC lines generated from diverse genetic backgrounds through global collaborative efforts. This is both an ethical obligation and a strategy that can be leveraged to gain insight into the contribution of variants that may differ across populations. Second, publications that clearly define and report the specific conditions utilized in hPSC studies that may contribute to variance in order to facilitate cross study evaluation, which could include the establishment or modification of reporting criteria in scientific journals that publish hPSC studies. Ideally, this would be coupled with studies focused on defining culture conditions that decrease unwanted sources of variance. Third, recognition that the genetic basis of ASD profoundly influences the appropriate use of hPSCs, with different scale required for tackling questions in syndromic versus nonsyndromic idiopathic disease, further dependent upon the specific differentiation paradigm utilized as well as the assay readout. As our understanding of ASD genetics continues to unfold, it will be critical for investigators to keep pace with power calculations that more accurately estimate the scale at which hPSC models should ideally be employed in order to draw conclusions from different genetic architectures, while utilizing additional strategies to reduce sample sizes to achieve more imminently realistic numbers. Fourth, continued advocacy for human fetal tissue research and investment in technologies to improve the fidelity of in vitro derived brain cell types that are most relevant for understanding ASD mechanisms. It is critical for the field to consider what improvements are essential for studying ASD, as opposed to what can be done to build the perfect “brain in a dish.” Fifth, recommendations from donors and their families, physicians, administrators, geneticists, and investigators for the design of future hPSC collection efforts. For example, the inclusion of ASD patient phenotyping data and genotyping/sequencing data that can be paired with family-based hPSC collections broadly consented for distribution and genomic data sharing to facilitate rational decisions about stratification of cell lines and data reproducibility. Several large consortium efforts are already underway, which require continued efforts to define, access, and distribute the correct information with the appropriate research subject protections. Importantly, all of these issues have come into sharper focus in the era of big data and developing solutions will improve our ability to decipher ASD mechanisms using hPSC models.

While it would be a mistake to claim that complex diseases of higher order cognition and function like ASD can be fully recapitulated in a dish, it is perhaps appropriate to argue that by using human genotypes that contain relevant molecular machinery, we can position ourselves to make discoveries with relevance to human disease, leveraging the noted strengths of this particular model system alongside thoughtful consideration of the current limitations. Specifically for ASD, we feel that hPSC models are best suited for the investigation of basic disease mechanisms and for the identification and evaluation of potential therapeutic targets through drug screening approaches. Based on current genetic data, upper-layer excitatory cortical neurons, inhibitory neurons, and microglia are promising candidate cell types for further study, with monolayer approaches currently yielding the most reproducible data. While a majority of ASD hPSC studies have focused on basic disease mechanisms (please see summaries included in Lee et al. and Wang et al. [9, 10]), a handful of studies have indeed utilized iPSC models for proof-of-concept drug screens in monogenic disease [89, 90] and we hope to see additional such studies in the future. While there are many challenges that must be overcome to move from ASD genetics into biological insight and actionable clinical information, hPSC resources remain a critically important partner alongside primary human tissue and in vivo animal models.

## Data Availability

Not applicable.

## Abbreviations

ASD: Autism spectrum disorder
CIRM: California Institute for Regenerative Medicine
CNV: Copy number variant
CV: Coefficient of variation
dbGAP: Database of Genotypes and Phenotypes
EBiSC: European Bank of induced pluripotent Stem Cells
EGA: European Genome-phenome Archive
ESC: Embryonic stem cell
FDR: False discovery rate
FMR1: Fragile X mental retardation 1
FXS: Fragile X syndrome
GDPR: General Data Protection Regulation
GDS: Genomic data sharing
GWAS: Genome-wide association study
HipSci: Human iPSC Initiative
hPSC: Human pluripotent stem cell
hPSCreg: European human pluripotent stem cell registry
iPSC: Induced pluripotent stem cell
IRB: Institutional Review Board
MTA: Material transfer agreement
NIA: National Institute on Aging
NIGMS: National Institute of General Medical Sciences
NIH: National Institutes of Health
NIMH: National Institute of Mental Health
NINDS: National Institute of Neurological Disorders and Stroke
QTL: Quantitative trait loci
SNV: Single nucleotide variant
WES: Whole exome sequencing
WGS: Whole genome sequencing
XCI: X chromosome inactivation
